# Germline Genetic Variation Modulates Tumor Progression and Metastasis in a Mouse Model of Neuroendocrine Prostate Carcinoma

**DOI:** 10.1371/journal.pone.0061848

**Published:** 2013-04-19

**Authors:** Shashank J. Patel, Alfredo A. Molinolo, Silvio Gutkind, Nigel P. S. Crawford

**Affiliations:** 1 Metastasis Genetics Section, Cancer Genetics Branch, National Human Genome Research Institute, National Institutes of Health, Bethesda, Maryland, United States of America; 2 Oral and Pharyngeal Cancer Branch, National Institute of Dental and Craniofacial Research, National Institutes of Health, Bethesda, Maryland, United States of America; Florida International University, United States of America

## Abstract

Neuroendocrine (NE) differentiation has gained increased attention as a prostate cancer (PC) prognostic marker. The aim of this study is to determine whether host germline genetic variation influences tumor progression and metastasis in C57BL/6-Tg(TRAMP)8247Ng/J (TRAMP) mouse model of aggressive NEPC. TRAMP mice were crossed to the eight progenitor strains of the Collaborative Cross recombinant inbred panel to address this. Tumor growth and metastasis burden were quantified in heterozygous transgene positive F1 male mice at 30 weeks of age. Compared to wild-type C57BL/6J-Tg(TRAMP)824Ng/J males, TRAMP x CAST/EiJ, TRAMP x NOD/ShiLtJ and TRAMP x NZO/HlLtJ F1 males displayed significant increases in tumor growth. Conversely, TRAMP x WSB/EiJ and TRAMP x PWK/PhJ F1 males displayed significant reductions in tumor growth. Interestingly, despite reduced tumor burden, TRAMP x WSB/EiJ males had an increased nodal metastasis burden. Patterns of distant pulmonary metastasis tended to follow the same patterns as that of local dissemination in each of the strains. All tumors and metastases displayed positive staining for NE markers, synaptophysin, and FOXA2. These experiments conclusively demonstrate that the introduction of germline variation by breeding modulates tumor growth, local metastasis burden, and distant metastasis frequency in this model of NEPC. These strains will be useful as model systems to facilitate the identification of germline modifier genes that promote the development of aggressive forms of PC.

## Introduction

Prostate cancer (PC) is the second leading cause of cancer mortality in men [1]. Despite the reduced mortality that has arisen with an increased availability of tools for early diagnosis and the success of hormone ablation therapies, advanced and recurrent PC remains incurable. Conventional assessment of PC prognosis relies heavily upon the Gleason grading scale to assess the histological severity of the primary tumor. However, this pathological grading system suffers from significant interobserver variability, and as a consequence, there is a need for additional means of assessing prognosis.

Neuroendocrine differentiation (NED), which is defined as the emergence of single or small clusters of neuroendocrine cells in conventional prostatic adenocarcinomas through trans-differentiation, has been gaining increasing attention as a potential prognostic marker [2]. Adenocarcinomas with significant NED are typically associated with a poorer prognosis, with NED being increased in high-grade and high stage tumors, and particularly in hormone treated and castration resistant disease [3]. Neuroendocrine prostate carcinomas (NEPC), which includes carcinoid tumor and small-cell carcinoma, are heterogeneous tumors containing mainly NE cells, and are associated with a poor outcome [2], [4]. Although NEPC are rare and comprise less than 2% of all tumors [5], [6], a sizable subset of conventional prostate adenocarcinomas displays NED. One plausible factor influencing the induction of NED is germline genetic variation. Despite the fact that recent studies have identified novel genes orchestrating this aggressive disease [2], [7], [8], it remains elusive that how host germline genetic variation influences susceptibility to NED or NEPC.

The influence of germline variation on tumor progression has been proven in other cancer types by introducing germline polymorphism into mouse models of cancer by breeding. This type of strategy was utilized in the FVB/N-Tg(MMTV-PyVT)634Mul/J mouse model of mammary tumorigenesis to identify progression and metastasis quantitative trait loci (QTLs). Subsequent fine mapping studies allowed for the characterization of *Brd4*, *Sipa1* and *Rrp1b* as novel breast cancer metastasis susceptibility genes [9], [10], [11], [12]. The clinical significance of these was established by demonstrating that germline polymorphisms within these genes are associated with poor prognosis in multiple human breast cancer cohorts [13], [14], [15].

The TRAMP transgenic mouse model was utilized in this study to investigate the role of host germline polymorphism in the development of aggressive prostate carcinoma. This model utilizes the androgen-responsive minimal probasin promoter (PB) to initiate SV40 virus T antigen (TAg)-mediated tumorigenesis in the prostatic epithelium. It is a particularly useful model for studying progression and metastasis, since by 30 weeks of age mice typically display a high incidence of multi-organ metastases, which is not a characteristic of any other prostate cancer mouse model [16], [17]. Also, studies have shown that NE phenotype tumors in TRAMP mice share certain key molecular features with NED and NEPC in humans, such as tumor growth regulation by high expression of AURKA, MYCN, FOXA2 and SIAH2 [7], [8], [18].

In this study, germline polymorphism was introduced by breeding to determine the extent to which host genetic variation influences tumor progression and metastasis in this model. Specifically, TRAMP mice were crossed to the eight progenitor strains of the Collaborative Cross (CC) recombinant inbred panel, which are a genetically diverse mapping panel that captures nearly 90% of the known variation present in laboratory mice [19], [20] This study conclusively demonstrates that the introduction of germline variation by breeding significantly modulates tumor growth, local metastasis burden, and distant metastasis frequency in TRAMP mice.

## Materials and Methods

### Animal Husbandry and Genotyping

C57BL/6J-Tg(TRAMP)824Ng/J (TRAMP-B6) mice and eight CC progenitor strains, C57BL/6J (B6), A/J (A), 129S1/SvImJ (129S), NOD/LtJ (NOD), NZO/HlLtJ (NZO), CAST/EiJ (CAST), PWK/PhJ (PWK) and WSB/EiJ (WSB), were obtained from Jackson Laboratories (Bar Harbor, ME). TRAMP-B6 females were crossed with eight CC progenitor strains to generate eight different F1 strains of mice (TRAMP-B6, TRAMP-A, TRAMP-129S, TRAMP-NOD, TRAMP-NZO, TRAMP-CAST, TRAMP-PWK and TRAMP-WSB, respectively) hemizygous for PB-TAg transgene. TRAMP-B6 females were bred with non-transgenic B6 males for colony maintenance. This study was carried out in strict accordance with the recommendations in the Guide for the Care and Use of Laboratory Animals of the National Institutes of Health. The protocol was approved by the National Human Genome Research Institute Animal Care and Use Committee (protocol number: G-09-2). All necropsies were performed under sodium pentobarbital anesthesia followed by cervical dislocation, and all efforts were made to minimize suffering. The mouse tail genomic DNA was extracted from F1 progeny with HotSHOT method [21] for genotyping analysis. To identify the hemizygous PB-TAg transgene positive F1 (TRAMP F1) mice, PCR screening was performed as described [22].

### Tissue Collection

TRAMP F1 male mice were sacrificed by pentobarbital overdose at 30 weeks of age or humane endpoint, whichever were achieved first. Humane experimental endpoints for this study were rapid weight loss, hunched posture, labored breathing, trauma, impaired mobility, dysuria, or difficulty in obtaining food or water. Prostatic tumor, seminal vesicles, lungs, liver, and lymph nodes were harvested from TRAMP F1 males. Prostatic tumor and seminal vesicles were weighed to quantify tumor burden. Tumor and seminal vesicle weights were normalized to age of the mouse at the time of euthanasia by multiplying the actual weight by the inverse ratio of the age in days at the time of death and the pre-designated experimental endpoint (i.e. 210 days). Visible enlarged lymph nodes in para-aortic region were weighed to quantify metastatic lymph node burden. These were again normalized to age of the mouse at the time of euthanasia. Lungs were collected to determine isolated tumor cell infiltrates in lung parenchyma and microscopic metastatic lesions. Other organs displaying macroscopic metastatic lesions through gross observation were also collected for histology. These collected tissues were fixed in buffered formalin (10% w/v phosphate buffered formaldehyde, Fisher Scientific) or Z-fix (zinc buffered formaldehyde, Anatech Ltd.) overnight and then transferred to 70% ethanol. Fixed tissues were embedded in paraffin, sectioned to a thickness of 4 µm and stained with hematoxylin and eosin (H&E). Histology slides were scanned with Scanscope Digital microscope (Aperio, Vista, CA).

### Immunohistochemistry

Tissues were collected and sectioned as described above for immunohistochemical (IHC) analyses. Histopathological analyses of these tissues were undertaken by Dr. A. Molinolo. Tissues were stained with the following primary antibodies: anti-synaptophysin, 1∶200 (Life Technologies, Carlsbad, CA); anti-cytokeratin 8, 1∶200 (CK8; ab59400, Abcam, Cambridge, MA); and anti-SV40 Large T-antigen (TAg), 1∶400, (BD Pharmingen, San Diego, CA). Paraffin blocks were sent to Center of Comparative Medicine, University of California, Davis, CA to perform IHC staining with anti-Foxa1 (C-20), 1∶1000 and anti-Foxa2 (P-19), 1∶1000, BD Transduction Laboratories, San Diego, CA.

### Time Course Experiments

TRAMP F1 males of B6, WSB, PWK and NOD backgrounds were allocated to the groups and separated throughout different time points. For each of these strains of TRAMP F1 mice, three mice were assigned for each time point. These mice were sacrificed at 4, 8, 12, 16, 20 and 25 weeks; prostate and seminal vesicles were harvested for histopathological analysis. Prostatic intraepithelial neoplasia (PIN) lesion in TRAMP mice has been termed as atypical hyperplasia of TAg in previous literature [23]. However, lesions of prostate epithelium were categorized into three categories, hyperplasia, PIN and carcinoma, following the conventional classification [4]. Prostates from these F1 males were collected at 8 weeks of age and subjected to IHC staining with anti-TAg to determine the strain specific differences in TAg expression in prostatic epithelium.

### Quantitative and Statistical Analysis

MedCalc (Mariakerke, Belgium) was used to analyze phenotype data. Statistical significance levels for observed differences were calculated with the Mann-Whitney U test. Kaplan-Meyer survival curves were plotted for survival analyses and hazard ratios calculated using the Cox proportional hazards model. Imagescope software (Aperio, Vista, CA) was used to quantify the nuclear expression of TAg in prostatic epithelium. The significance levels for the strain specific differences in TAg expression were determined with Fisher’s exact test. Correlation coefficient and level of its significance were equated with Spearman’s rank analysis. *P<*0.05 was considered significant.

## Results

### Germline Genetic Variation Modulates Tumor Associated Mortality and Tumor Burden in TRAMP Mice

**Figure 1:**
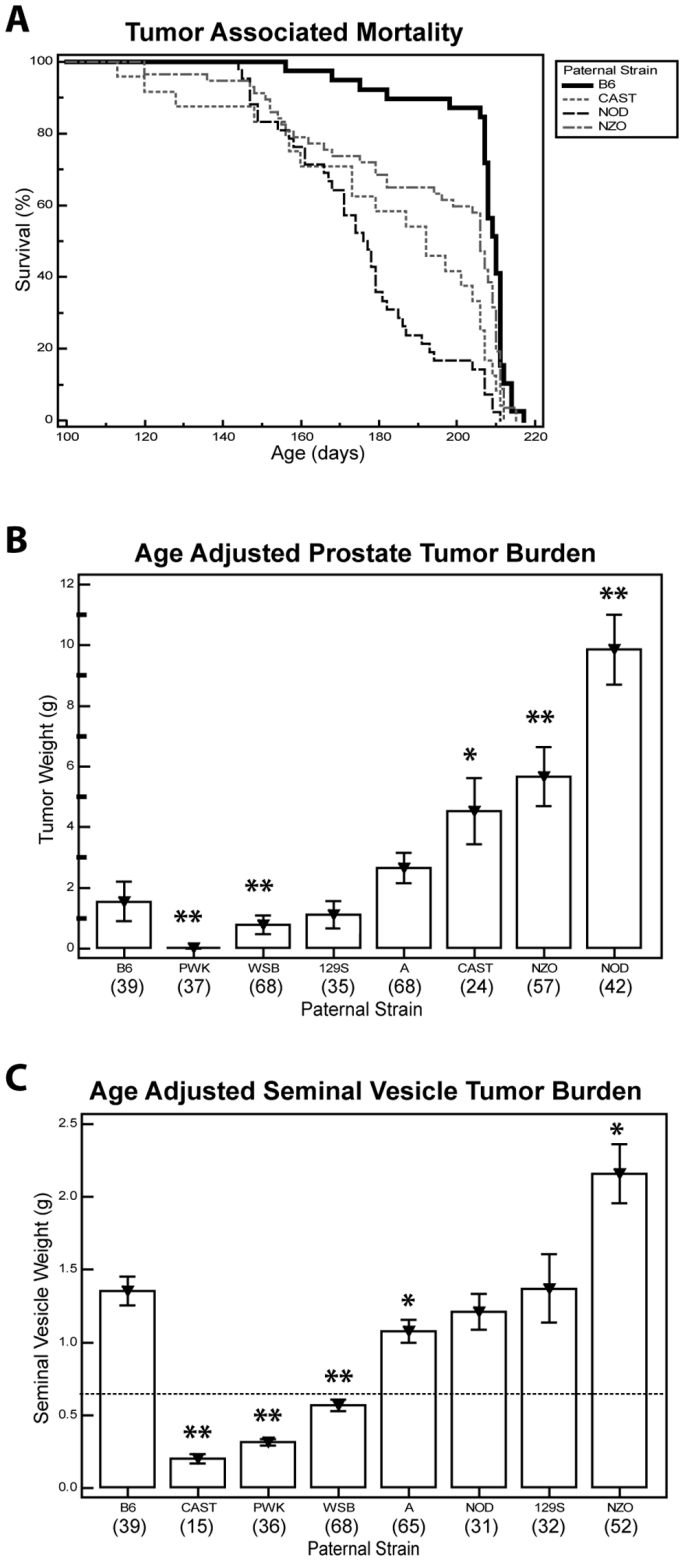
Genetic background influences prostate and seminal vesicle tumor burdens, and tumor associated mortality in TRAMP mice. (a) Kaplan-Meier survival curve for TRAMP F1 strains with a significantly increased tumor-associated mortality compared to wildtype C57BL/6J-Tg(TRAMP)824Ng/J (B6) mice. (b) Average age adjusted prostate tumor burden in TRAMP F1 strains. (c) Average age adjusted seminal vesicle tumor burden in TRAMP F1 strains. Dotted line at 0.6 g on *y*-axis represents the maximum weight of seminal vesicles recorded in transgene negative TRAMP F1 mice at 30 weeks of age. Bar graphs represent average weight of prostate tumor or seminal vesicles ± SEM. *P<0.050 and **P<0.001. Values in parentheses below *x*-axis represent the number of animals evaluated in each group.

The aim was to assess tumor growth and metastasis in TRAMP F1 males at 30 weeks of age. However this goal was frequently not achievable owing to factors relating to the overall health of the mouse necessitating euthanasia for humane reasons listed above. Tumor-associated mortality was therefore followed for TRAMP F1 mice, and significant differences were observed in a number of the F1 strains (Fig. 1a). Compared to wildtype C57BL/6J-Tg(TRAMP)824Ng/J males, tumor associated mortality was significantly higher in TRAMP-NOD (hazard ratio [HR] = 2.26 [1.76–2.92]; *P*<0.001), TRAMP-CAST (HR = 3.03 [1.77–5.16]; *P*<0.001) and TRAMP-NZO (HR = 1.18 [1.02–1.35]; *P* = 0.022). TRAMP-A, TRAMP-129S, TRAMP-WSB and TRAMP-PWK males did not show any significant difference in their survival times in comparison to wildtype C57BL/6J-Tg(TRAMP)824Ng/J males.

To account for differences in tumor associated mortality, tumor weights were adjusted in all strains to account for age of the mouse at the time of euthanasia/death. Compared to wildtype C57BL/6J-Tg(TRAMP)824Ng/J males (age adjusted mean ± SEM of tumor burden = 1.54±0.65 g, *n* = 35), both TRAMP-NOD (*n* = 42) and TRAMP-NZO (*n = *57) males displayed a significant increase in tumor growth (tumor burden = 9.86±1.16 g and 5.66±0.97 g, respectively; *P<*0.001; Fig. 1b). TRAMP-CAST animals (*n = *24) also displayed a significant increase in tumor growth, albeit at a somewhat lesser level of significance (tumor burden = 4.53±1.09 g; *P = *0.050; Fig. 1b). Conversely, both TRAMP-WSB (*n = *68) and TRAMP-PWK (*n = *37) males displayed a significant reduction in tumor growth (tumor burden = 0.79±0.31 g [*P* = 0.001] and 0.02±0.01 g [*P<*0.001], respectively; Fig. 1b). Indeed, only 29/68 TRAMP-WSB mice and 1/37 TRAMP-PWK mice developed macroscopic tumors, weighing ≥0.2 g. TRAMP-129S (*n = *35) and TRAMP-A (*n = *68) did not show significant difference in tumor growth compared to wild-type TRAMP mice. These data conclusively demonstrate that the introduction of germline variation by breeding significantly modulated tumor burden in the TRAMP mouse.

As was the case in the previous studies [24], [25], seminal vesicle tumors were observed in TRAMP F1 males. Seminal vesicle weight was therefore recorded as an indicator of this type of tumor growth. Seminal vesicles from TRAMP-CAST (*n = *15), TRAMP-PWK (*n = *37), TRAMP-WSB (*n = *68) and TRAMP-A (*n = *65) showed significantly less tumor growth (age adjusted seminal vesicle burden = 0.20±0.03 g, 0.31±0.02 g, 0.57±0.04 g and 1.08±0.08 g respectively; *P<*0.020) compared to wildtype C57BL/6J-Tg(TRAMP)824Ng/J males (seminal vesicle burden = 1.36±0.10 g, *n = *39; Fig. 1c). However, comparatively, TRAMP-NZO displayed significantly higher tumor growth (seminal vesicle burden = 2.16±0.04 g, *n = *52; *P = *0.009; Fig. 1c).

### Germline Variation does not Affect TAg Expression in Prostatic Epithelium

The dramatic reduction in prostate tumor burden seen in the TRAMP-PWK and TRAMP-WSB strains compared to other F1 mice could be due to suppression of TAg transgene in the prostatic epithelium. To eliminate this possibility, IHC analysis for TAg transgene expression was performed using prostates from 8 week old wildtype C57BL/6J-Tg(TRAMP)824Ng/J, TRAMP-PWK, TRAMP-WSB and TRAMP-NOD mice. The percentage of prostatic acinar cells expressing TAg from anterior, lateral, ventral and dorsal lobes was quantified. The total amount of cells expressing TAg in the prostate did not vary significantly in TRAMP-PWK, TRAMP-WSB and TRAMP-NOD mice compared to wildtype C57BL/6J-Tg(TRAMP)824Ng/J (*P>*0.1; Fig. S1). These data indicate that TAg expression does not differ among strains and that the observed phenotypic differences are due to germline genetic variation.

### Strain Specific Differences in Prostate Carcinoma Metastasis

**Figure 2:**
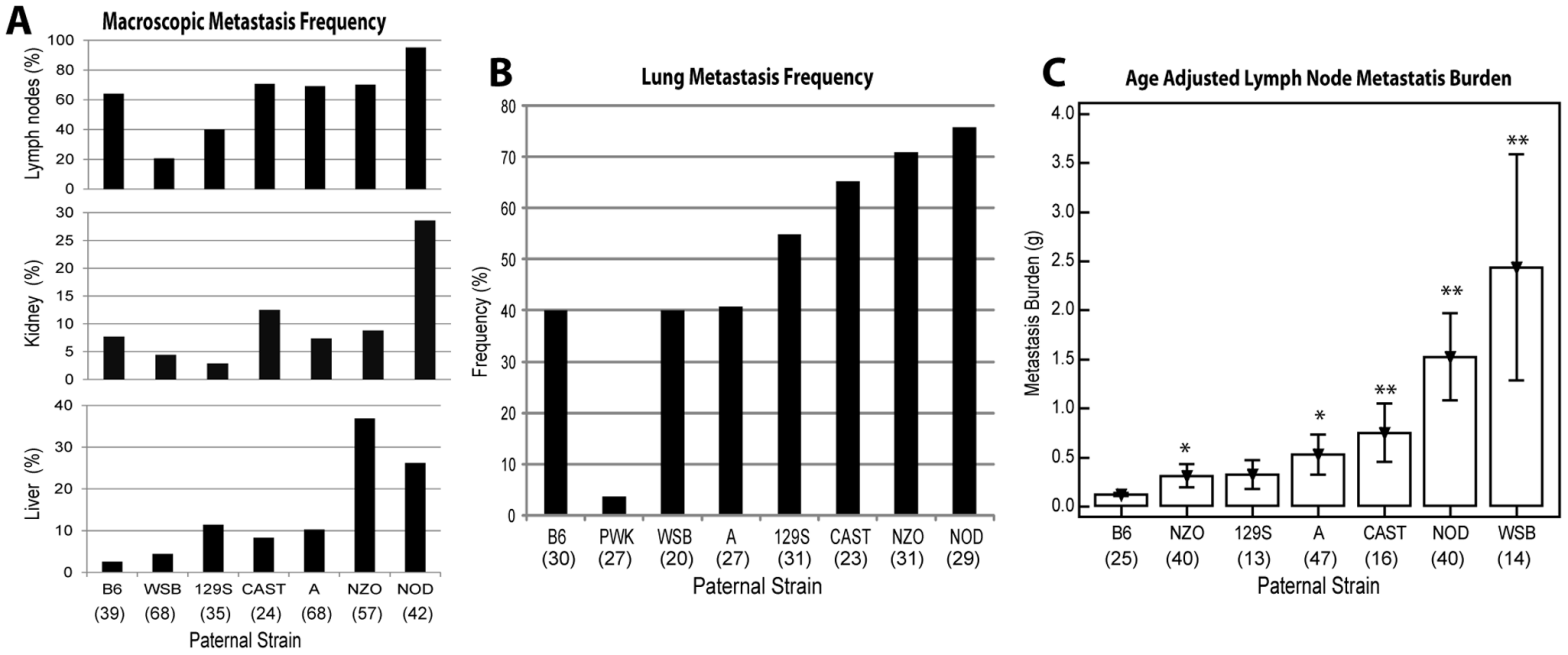
Effect of paternal genotype on metastatic efficiency of carcinoma cells in TRAMP mice. (**a**) Incidence of macroscopic metastases in lymph nodes, kidney and liver modulated by paternal genotype. Data not shown for TRAMP-PWK since macroscopic metastases were not observed in this strain. (**b**) Incidence of lung metastases modulated by paternal genotype. These incidence counts include the presence of isolated NE tumor cells, microscopic or macroscopic metastases in lung parenchyma. (**c**) Average age adjusted lymph node metastasis burden affected by paternal genotype. The total nodal metastasis burden for less enlarged lymph nodes (<0.1 g) was rounded to 0.1 g. Bar graphs represent average nodal burden ± SEM. *P<0.050 and **P<0.001 for comparisons *vs.* wildtype C57BL/6J-Tg(TRAMP)824Ng/J (B6). Data not shown for TRAMP-PWK since nodal metastases were not observed in this strain. Values in parentheses below *x*-axis represent the number of animals evaluated in each group.

Prostate carcinoma in the TRAMP model exhibits multi-organ metastasis, primarily to lymph nodes and also to lungs, liver, kidney and bones [16]. In order to examine the effect of strain-specific modifier loci on tumor cell capacity to disseminate to distant organs via lymphatic and blood vessels, the incidence of metastasis were tracked in pelvic and renal lymph nodes, lungs, liver and kidney in TRAMP F1 mice. Strain-specific variation was observed in the incidence of metastases in multiple organs (Fig. 2a and 2b). IHC for SV40-TAg and androgen receptor were performed to better characterize these lesions. Regardless of strain background, all metastases stained positive for SV40-TAg and negative for AR (Fig. S2).

A high incidence of metastasis was observed in some F1 strains. For example, 40/42 (95%) of TRAMP-NOD males developed metastases in local lymph nodes, 12/42 (29%) developed liver metastases, and 22/29 (75%) developed pulmonary metastases. Conversely, a low incidence of metastasis was observed in other strains. For example, only 14/68 (21%) TRAMP-WSB males developed nodal metastases, 8/41 (20%) pulmonary metastases and 3/41 (4%) hepatic metastases.

Several of the F1 strains exhibited striking differences in age adjusted local nodal metastasis burden (Fig. 2c) compared to the actual frequency of nodal metastasis (Fig. 2a). The wildtype C57BL/6J-Tg(TRAMP)824Ng/J strain exhibited the lowest age adjusted local metastasis burden with an average local nodal weight of 0.12±0.02 g. Five F1 strains had a significantly increased age adjusted local nodal metastasis compared to this: TRAMP-NZO (met burden = 0.31±0.12; *P* = 0.012), TRAMP-A (met burden = 0.53±0.20; *P* = 0.049), TRAMP-CAST (met burden = 0.75±0.30; *P*<0.0001), TRAMP-NOD (met burden = 1.53±0.45; *P*<0.0001) and TRAMP-WSB (met burden = 2.44±1.15; *P* = 0.002).

### Characterization of the NE Phenotype in TRAMP Tumors

**Figure 3:**
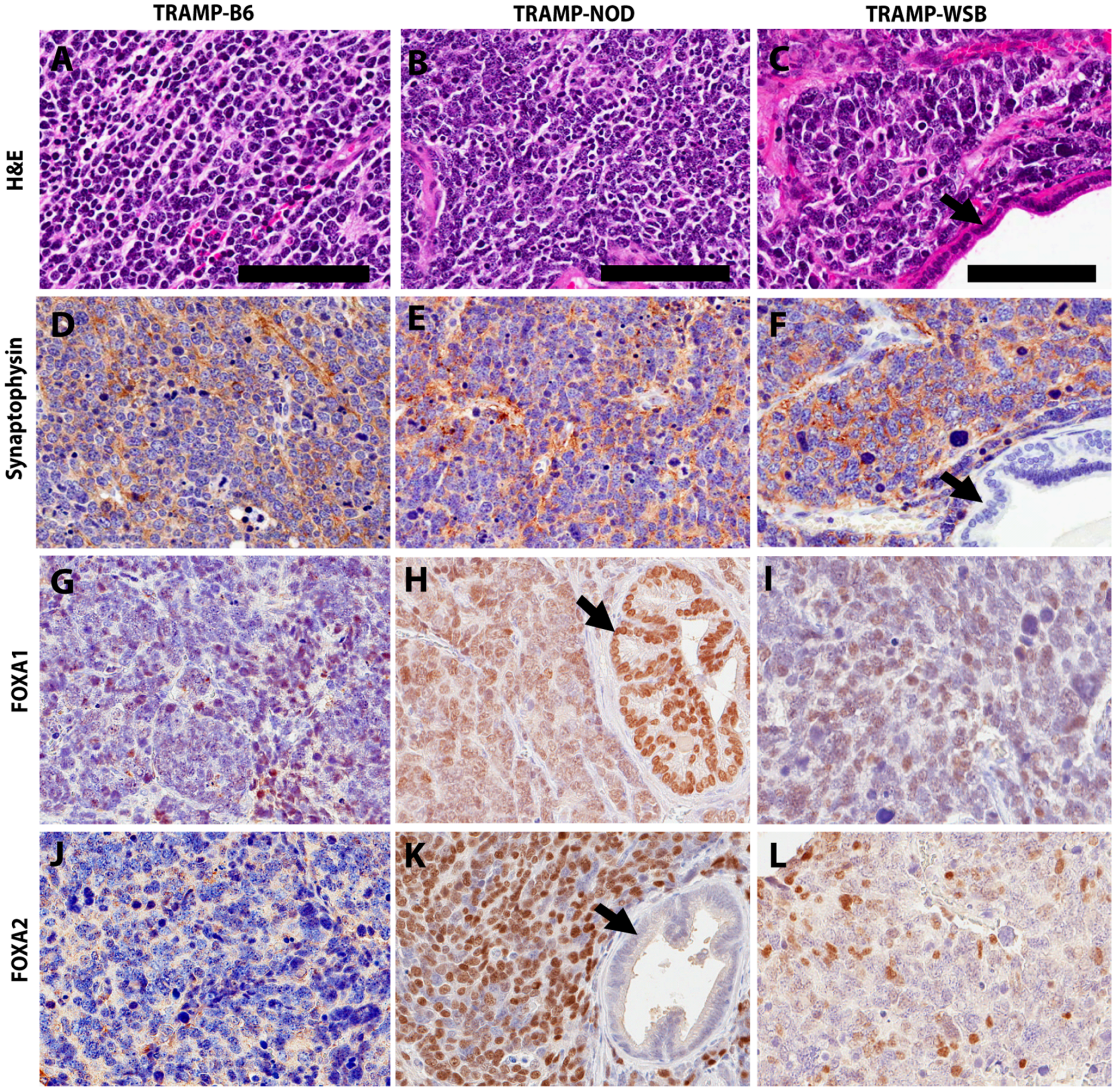
Representative photomicrographs of strain-specific differences in the expression of NE markers in prostate tumors from TRAMP F1 mice. Panels (a), (b) and (c) show H&E staining of the tumors; Panels (d), (e) and (f) show IHC staining of tumors with NE marker synaptophysin; Panels (g), (h) and (i) show IHC staining with FOXA1; Panels (j), (k) and (l) show IHC staining with FOXA2. Arrows point to normal prostate luminal cells, showing positive staining for FOXA1 and no staining for the NE markers, synaptophysin and FOXA2. Bar denotes 100 µm.

**Figure 4:**
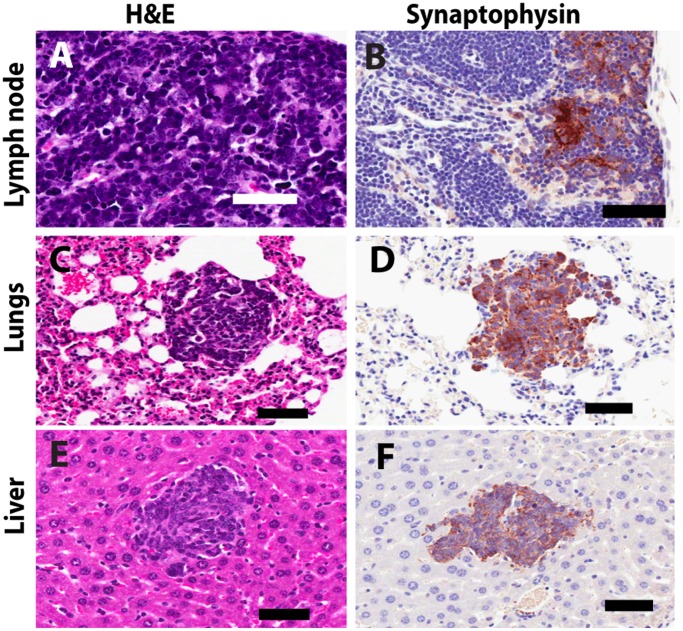
Representative photomicrographs of metastatic lesions displaying NE phenotype in organs harvested from TRAMP F1 mice. Panels (a), (c) and (e) show H&E staining of the metastatic lesions in the lymph node, lungs and liver, respectively. Panels (b), (d) and (f) show strong positive IHC staining of metastatic PC cells with synaptophysin. Bar denotes 50 µm.

NE phenotype prostate tumor cells in TRAMP mice display similar morphological and molecular characteristics to human NED and NEPC cells, including the expression of IHC markers, synaptophysin, chromogranin A and FOXA2, and loss of expression of cytokeratin 8 (CK8) [2], [4], [7], [8], [26]. IHC for the NE marker synaptophysin was performed in tumors and metastatic lesions. Regardless of strain background, all macroscopic tumors (≥0.2 g) stained positive for synaptophysin (Fig. 3). Tumor cells infiltrating lung, liver and lymph node tissues also stained positive for synaptophysin (Fig. 4). These observations establish that tumor burden and nodal metastatic burden, used as quantitative traits in this study, represent phenotypes of NEPC.

To further characterize the NE phenotype, IHC was performed for the transcription factors FOXA1 and FOXA2, and the luminal epithelial marker CK8. As expected, CK8 staining was absent in all TRAMP F1 tumors (data not shown). Staining for FOXA2 mirrored that of synaptophysin, and was observed in all tumors. However, such consistency was not observed when comparing FOXA1 and FOXA2 staining patterns with some foci staining strongly for both FOXA1 and FOXA2, while others stained strongly for only either one of these transcription markers (Fig. 3 g–l). Such differences in FOXA1 and FOXA2 staining were not strain-dependent and most likely represent heterogeneity of the overall process of tumorigenesis.

### Germline Factors Mediate NE Carcinoma Initiation

To investigate differences in tumor initiation and progression, serial histological analysis of prostate and seminal vesicles was performed in some of the strains with the most divergent phenotypes compared to the wildtype C57BL/6J-Tg(TRAMP)824Ng/J strain (TRAMP-NOD, TRAMP-WSB and TRAMP-PWK). Hyperplastic lesions were evident in dorsolateral and anterior prostates in all strains as early as 4 weeks. By 8 weeks of age, these hyperplastic lesions invariably progressed to PIN. The severity and number of hyperplastic foci and PIN lesions increased with age in these F1 mice, and no strain differences were noted.

Seminal vesicle hyperplasia was benign in nature and progressed to become papillary adenomas or phyllodes-like tumor consisting of stromal and epithelial proliferation protruding into the glandular lumen. NE carcinomas consist of solid masses of tumor cells with atypical and pleomorphic cells without nuclear polarity and scant cytoplasm. The histological features of NE carcinoma and seminal vesicle hyperplasia did not differ between wildtype C57BL/6J-Tg(TRAMP)824Ng/J, TRAMP-NOD, TRAMP-WSB and TRAMP-PWK strains. Similarly, histological features did not differ from wildtype C57BL/6J-Tg(TRAMP)824Ng/J mice in all other F1 strains studied (data not shown). NE carcinomas were found to be arising from ventral and/or dorsolateral lobes of prostate in TRAMP mice. These strains exhibited differences in the incidence of seminal vesicle hyperplasia and NE carcinomas (Table S1).

The earliest time point when the NE lesion was evident in TRAMP-NOD mice was 8 weeks. The incidence of NE carcinoma increased with age, and by 16 weeks, 100% of TRAMP-NOD mice (6/6) displayed NE carcinoma lesions or tumor. Conversely, only 1 out of 15 wildtype C57BL/6J-Tg(TRAMP)824Ng/J males developed NE tumor at 16 weeks of age. In our time-course study, all TRAMP-WSB mice developed seminal vesicle hyperplasia by 25 weeks of age (6/6), but none had NE carcinoma. Given that no tumors were evident in TRAMP-PWK mice by 30 weeks of age, a set of these mice were aged to 50 weeks (*n* = 4). Interestingly, only 1 of these TRAMP-PWK mice developed NE tumors and died at 38 weeks of age. Another TRAMP-PWK mouse developed only hyperplasia, and 2 of these 4 TRAMP-PWK developed severe hyperplasia and phyllodes-like proliferations at 50 weeks. These data emphasize previous observations that NE lesions arise independently of the PIN lesions in the TRAMP model [23]. More importantly, the observed strain-specific differences demonstrate that paternal genotypes substantially affect the initiation and kinetics of NE tumorigenesis in TRAMP F1 mice.

## Discussion

Although NE carcinoma represents only 1–2% of prostate malignancies, this form of PC is highly aggressive and has a poor survival rate [3], [27]. Additionally, NED is present in almost >90% of high grade adenocarcinomas of prostate [28]. More importantly, androgen depletion therapy strongly stimulates NED [29], [30]. The characteristics of these types of tumor, including aggressive tumor growth, multi-organ metastases and relapse of disease after castration or androgen depletion are well recapitulated by TRAMP mouse model [24], [31], [32]. The overall aim of this work is to identify germline susceptibility genes that are associated with an increased risk of NED development. The potential clinical significance of this is that it may prove possible to type polymorphic germline modifier genes at the time of PC diagnosis to more accurately identify men at risk of progression, and thus initiate more aggressive therapies in these metastasis-prone individuals.

Germline predisposition to diseases displaying a complex, non-Mendelian inheritance pattern is controlled by polymorphisms within multiple genes, each of which likely have a small effect in isolation [33]. The identification of modifier genes of tumor progression and metastasis has proven particularly difficult using genome wide and familial association studies. An alternate approach has been to map disease modifier loci using mouse models of human disease [34], [35]. Selection of the appropriate mouse strain(s) for this type of study is of critical importance to maximize genetic power and increase the likelihood of identifying genes relevant to human disease [36], [37]. This study was carried out to determine whether germline modifiers modulate the development of aggressive NEPC in TRAMP model. Our data have proven that this is indeed the case, and the information gained from these analyses will be used to direct future genetic analyses.

In this study, polymorphisms carried by NOD males were shown to increase tumor growth and dissemination. Conversely, progression was significantly suppressed by modifiers inherited from PWK males, with NE tumorigenesis being almost completely suppressed in this strain. A particularly interesting observation was that TRAMP-CAST and TRAMP-WSB strains displayed a particularly high propensity for metastasis, where the nodal metastasis burden frequently exceeded the primary tumor weight (Fig. 2C). Indeed, the disease course in the TRAMP-WSB strain was particularly unusual when one considers that only 29/68 developed macroscopic tumorigenesis and that the overall age adjusted prostate and seminal vesicle tumor burden in this strain was significantly lower than wildtype C57BL/6J-Tg(TRAMP)824Ng/J mice (see Fig. 1a and 1b, respectively). The dissociation of tumor growth and metastasis in this strain underscores the differing mechanisms involved in these two processes and makes TRAMP-WSB a particularly important strain for future genetic mapping studies.

Additionally, our time-course experiment data showed that prostate and seminal vesicle tumor initiation are controlled by inherited polymorphic genes in these strains. This time-course study demonstrated that there is dissociation among the experimental groups between the development of neuroendocrine prostate tumors, which are high in the TRAMP-NOD strain but low in the other groups and seminal vesicle hyperplasia, which is high in the wildtype C57BL/6J-Tg(TRAMP)824Ng/J and TRAMP-WSB strains but low in TRAMP-NOD and TRAMP-PWK. Overall, these data imply that these differing components of tumorigenesis in TRAMP mice are influenced by different germline polymorphisms. Interestingly, another study reported that NE carcinoma occurs in 20% of TRAMP mice on B6 background and 100% of mice on FVB background, and in ∼50% of TRAMP-B6 x FVB F1 mice. This suggests that the interaction of modifiers present in B6 mice decreased NEPC incidence compared to TRAMP-FVB mice [23]. However, the present study demonstrates that modifiers present in NOD mice can override the suppressive effect of B6 strain modifiers to promote NEPC incidence. Collectively, the presented data demonstrate that polymorphic genes derived from paternal strains likely influence tumor progression at multiple stages of disease development.

We do however acknowledge that the TRAMP model suffers a number of limitations. Specifically, the histological features of TRAMP tumors are much more reminiscent of human NEPC, which is relatively rare in humans, rather than the far more common adenocarcinoma with NED. The precise origins of the neoplastic process in the TRAMP mouse were clearly described in the seminal work of Chiaverotti et. al. [23]. This study determined that two distinct neoplastic cellular lineages evolve within TRAMP tumors. The first lineage, which was termed ‘abnormal hyperplasia of TAg’, was determined to follow a benign course and does not progress beyond pre-malignant PIN lesions. The second lineage, which accounts for the aggressive malignancies observed in TRAMP mice, arises spontaneously and has a strong neuroendocrine phenotype. Our work confirms the findings of this important study. Specifically, although abnormal hyperplasia of TAg and PIN was observed in all F1 animals, the incidence or kinetics of these did not differ between strains. As observed by Chiaverotti et. al. [23], strain differences appear to exert the majority of their influence upon the NE component of TRAMP tumorigenesis. Another limitation of this model is the relative infrequency of skeletal metastases [16]. Initial analyses demonstrated a slight increase in the frequency of metastases to long bones in more F1 mice displaying a more aggressive phenotype (e.g. TRAMP-NOD). However, the overall incidence of bone metastases remained low and it is unlikely that formal quantification would yield any data of value in the cohort sizes described here.

Regardless of the limitations of the TRAMP model, we argue that the modifier genes identified in future studies performed on the basis of the experiments performed here are likely to be applicable not only to NEPC but also more generally to the pathogenesis of aggressive human PC. Prostate adenocarcinomas and NE carcinomas derived from both human and mice all possess the capability to metastasize to lymph nodes, lungs and liver. This indicates that these two distinct tumor cell types may well have acquired similar molecular characteristics under the influence of common allelic determinants to promote a more metastatic phenotype. This is supported by the fact that metastatic disease is enhanced in recurrent PC after androgen depletion therapy, which is similar to the highly metastatic behavior displayed by NEPC cells lacking androgen receptors [38]. In addition, both androgen-independent PC and NEPC cells over-express transcription factors such as FOXA1, indicating the involvement of common factors in the transcriptional regulation in these cells [39], [40], [41]. These observations support the notion that common pathways may well be involved in the modulation of adenocarcinoma-associated NED and NE prostate cancer progression.

In conclusion, this study establishes that germline polymorphisms are significant modulators of NE tumor initiation and growth kinetics in the TRAMP model. TAg-mediated tumorigenesis and metastasis have been characterized in the progenitor strains of the CC recombinant inbred panel, which will facilitate future modifier mapping studies. Identification of the candidate genes and characterization of genetic polymorphisms associated with this aggressive form of prostate disease will facilitate an improved understanding of this aspect of PC pathogenesis and potentially facilitate the identification of individuals at increased risk of NED. Additionally, selective use of these TRAMP F1 strains in preclinical chemotherapeutic trials for specifically targeting metastasis or tumor growth of NEPC may prove to be a valuable tool.

## Supporting Information

Figure S1 **Strain specific differences in the expression of TAg transgene in prostate epithelia of TRAMP F1 mice.** Transgene was found to be expressing predominantly in ventral and dorsolateral prostates (representative photomicrographs **(a), (b)** and **(c)**) of TRAMP F1 mice. **(c)**. Percentage of cells showing positive IHC staining with anti-TAg across different strains of F1 mice from ventral, dorsal, lateral and anterior lobes of prostate collected from 8 weeks old TRAMP F1 mice. Bar graph represents average percent of cells stained positive ± SD for TRAMP F1 strains. Bar denotes 50 µm.
(TIF)

Figure S2 **Representative IHC analysis of TRAMP F1 metastatic lesions.** IHC analysis for androgen receptor (AR) and SV40-TAg was performed to better characterize metastatic lesions in TRAMP F1 strains. With regards to AR, immunostaining was positive in a high proportion of the nuclei in a paraffin embedded section of normal prostate **(a)**. No staining was seen in either lymph nodes **(c)** or lung metastases **(e)**, regardless of strain background. For the SV40-TAg, the normal prostate control is not immunoreactive **(b)**, but strong positivity was seen in the nuclei of both lymph nodes **(d)** and lung metastases **(f)**. The arrows point to the metastatic area embedded in the lung parenchyma. Bar denotes 50 µm.
(TIF)

Table S1 Strain related variation in pathological progression of NE carcinoma. Histopathological analysis of prostates from those F1 strains displaying greatest phenotypic variability compared to TRAMP-B6 was performed at various different ages. The number of mice in this time course experiment with evidence of various neoplastic lesions for each strain is displayed in the table.
(XLS)
