# Supplementary figures and images for: Germline Genetic Variation Modulates Tumor Progression and Metastasis in a Mouse Model of Neuroendocrine Prostate Carcinoma
Source: PLoS One. 2013 Apr 19;8(4):e61848. doi: 10.1371/journal.pone.0061848 (PMC3631138; doi:10.1371/journal.pone.0061848)

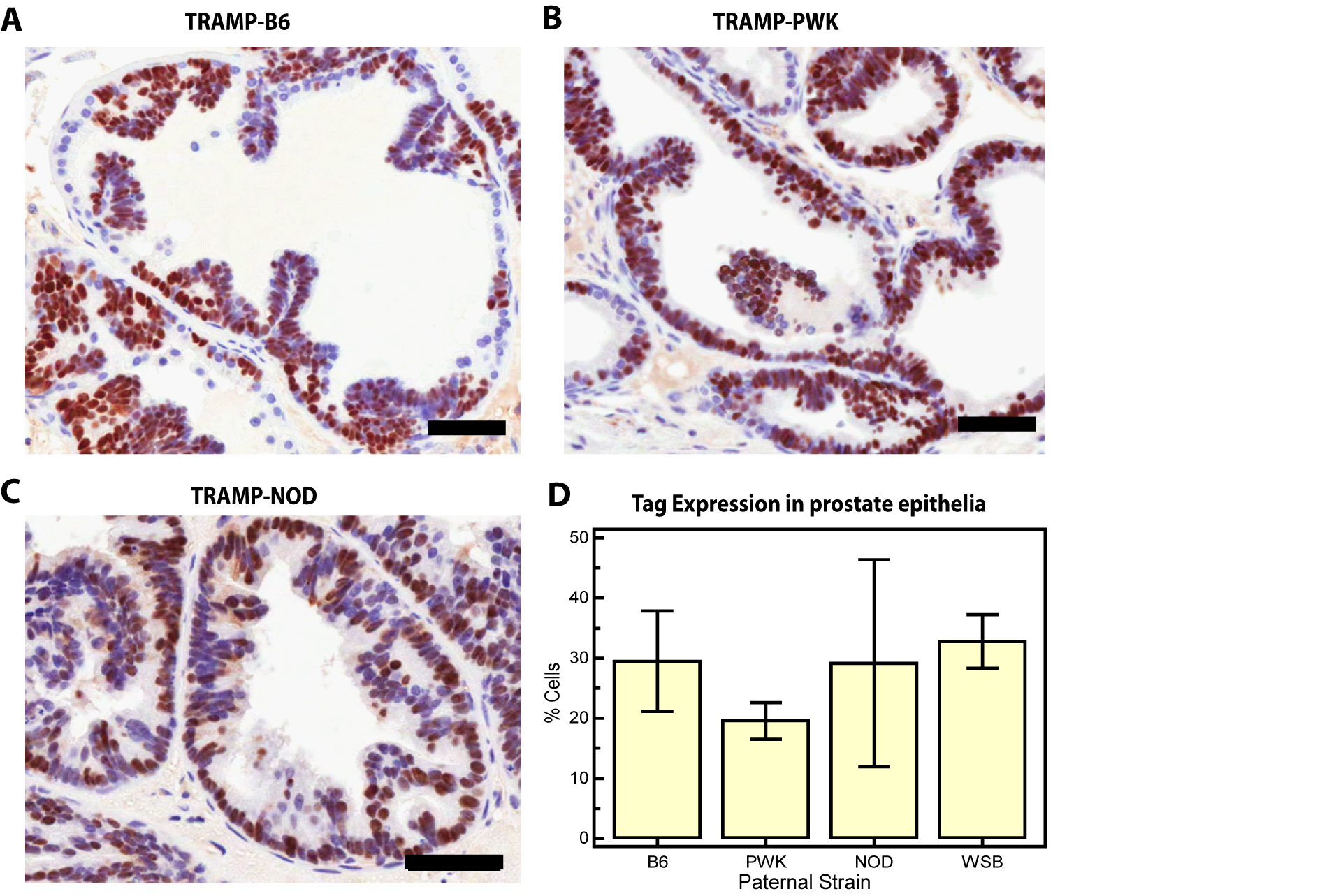

Supplement: Figure S1 — Strain specific differences in the expression of TAg transgene in prostate epithelia of TRAMP F1 mice. Transgene was found to be expressing predominantly in ventral and dorsolateral prostates (representative photomicrographs (a), (b) and (c)) of TRAMP F1 mice. (c). Percentage of cells showing positive IHC staining with anti-TAg across different strains of F1 mice from ventral, dorsal, lateral and anterior lobes of prostate collected from 8 weeks old TRAMP F1 mice. Bar graph represents average percent of cells stained positive ± SD for TRAMP F1 strains. Bar denotes 50 µm. (TIF) [file pone.0061848.s001.tif]

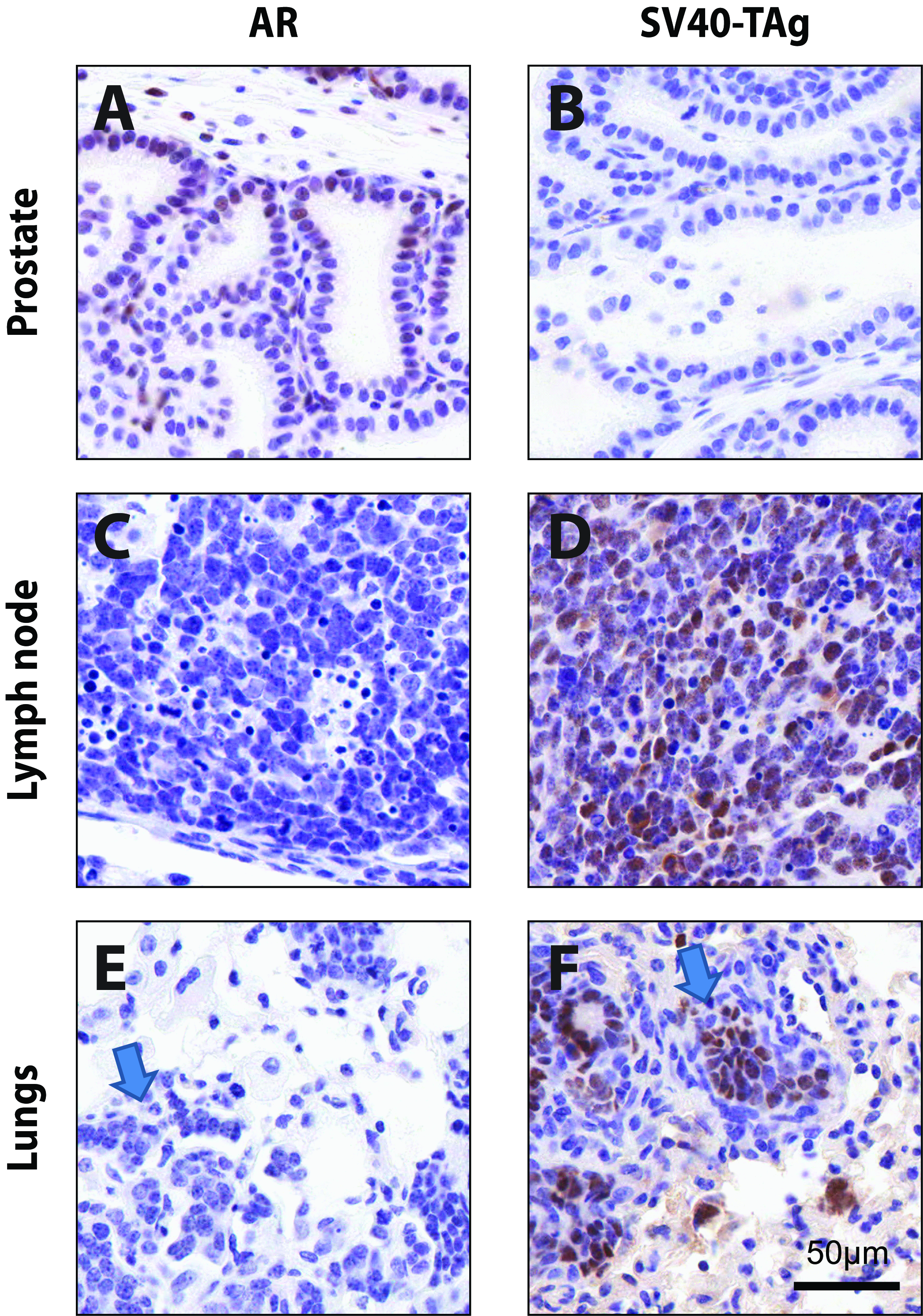

Supplement: Figure S2 — Representative IHC analysis of TRAMP F1 metastatic lesions. IHC analysis for androgen receptor (AR) and SV40-TAg was performed to better characterize metastatic lesions in TRAMP F1 strains. With regards to AR, immunostaining was positive in a high proportion of the nuclei in a paraffin embedded section of normal prostate (a). No staining was seen in either lymph nodes (c) or lung metastases (e), regardless of strain background. For the SV40-TAg, the normal prostate control is not immunoreactive (b), but strong positivity was seen in the nuclei of both lymph nodes (d) and lung metastases (f). The arrows point to the metastatic area embedded in the lung parenchyma. Bar denotes 50 µm. (TIF) [file pone.0061848.s002.tif]
